# HDNLS: Hybrid Deep-Learning and Non-Linear Least Squares-Based Method for Fast Multi-Component T1ρ Mapping in the Knee Joint

**DOI:** 10.3390/bioengineering12010008

**Published:** 2024-12-25

**Authors:** Dilbag Singh, Ravinder R. Regatte, Marcelo V. W. Zibetti

**Affiliations:** Center of Biomedical Imaging, Department of Radiology, New York University Grossman School of Medicine, New York, NY 10016, USA; ravinder.regatte@nyulangone.org

**Keywords:** multi-component MRI fitting, non-linear least squares (NLS), T1ρ mapping, deep learning, knee joint

## Abstract

Non-linear least squares (NLS) methods are commonly used for quantitative magnetic resonance imaging (MRI), especially for multi-exponential T1ρ mapping, which provides precise parameter estimation for different relaxation models in tissues, such as mono-exponential (ME), bi-exponential (BE), and stretched-exponential (SE) models. However, NLS may suffer from problems like sensitivity to initial guesses, slow convergence speed, and high computational cost. While deep learning (DL)-based T1ρ fitting methods offer faster alternatives, they often face challenges such as noise sensitivity and reliance on NLS-generated reference data for training. To address these limitations of both approaches, we propose the HDNLS, a hybrid model for fast multi-component parameter mapping, particularly targeted for T1ρ mapping in the knee joint. HDNLS combines voxel-wise DL, trained with synthetic data, with a few iterations of NLS to accelerate the fitting process, thus eliminating the need for reference MRI data for training. Due to the inverse-problem nature of the parameter mapping, certain parameters in a specific model may be more sensitive to noise, such as the short component in the BE model. To address this, the number of NLS iterations in HDNLS can act as a regularization, stabilizing the estimation to obtain meaningful solutions. Thus, in this work, we conducted a comprehensive analysis of the impact of NLS iterations on HDNLS performance and proposed four variants that balance estimation accuracy and computational speed. These variants are Ultrafast-NLS, Superfast-HDNLS, HDNLS, and Relaxed-HDNLS. These methods allow users to select a suitable configuration based on their specific speed and performance requirements. Among these, HDNLS emerges as the optimal trade-off between performance and fitting time. Extensive experiments on synthetic data demonstrate that HDNLS achieves comparable performance to NLS and regularized-NLS (RNLS) with a minimum of a 13-fold improvement in speed. HDNLS is just a little slower than DL-based methods; however, it significantly improves estimation quality, offering a solution for T1ρ fitting that is fast and reliable.

## 1. Introduction

Quantitative magnetic resonance imaging (qMRI) is essential for accurately evaluating tissue properties and detecting early pathological changes across various anatomical regions before the development of morphological changes [1,2,3]. Traditional qualitative MRI imaging, used in clinical practice, often lacks the sensitivity and specificity required for such early detection [4]. qMRI overcomes this problem by acquiring multi-contrast images with controlled variations in MRI pulse-sequence parameters [5]. It enables the estimation of tissue relaxation properties, and non-linear least squares (NLS) methods are usually the reference approaches to compute quantitative parameters [6,7,8].

NLS methods are fundamental tools in inverse problems and statistical estimations, particularly in parameter fitting. These methods extend linear regression to handle problems where the variables have a non-linear relationship. They aim to minimize the sum of squared differences between observed and predicted values [9,10]. The theoretical support and soundness of NLS methods, such as Jacobian-based approaches (like the Gauss–Newton and Levenberg–Marquardt approaches) and derivative-free algorithms (like the Nelder–Mead method) are strong, making them the preferred choice in multiple applications, but they are generally slow for large multi-dimensional datasets, among other problems [11,12,13].

Gauss–Newton methods are efficient for those problems where models are nearly linear. To achieve better convergence speed, it utilizes the Jacobian matrix to approximate the Hessian [9,10]. The Levenberg–Marquardt algorithm refines the Gauss–Newton method by using the concept of a damping factor. Thus, it enhances convergence stability and performance in more complex non-linear problems when the Hessian is ill-conditioned [11,12]. Powell’s Dog Leg method integrates Gauss–Newton and the steepest descent directions to effectively manage substantial curvature in the error surface. In contrast, hybrid approaches that combine the Levenberg–Marquardt algorithm with Quasi-Newton methods use adaptive step sizes and approximate Hessians to achieve robustness and efficiency in large-scale optimization problems [9,10,11]. Approximation-based versions of these methods, (i.e., adaptations of the Levenberg–Marquardt and Dog Leg approaches) replace exact derivative computations with approximations [12,13]. This substitution reduces computational complexity and improves efficiency [14,15]. Trust Region Conjugate Gradient (TRCG) [9] combines trust-region methods with iterative inversion of Hessian or its approximation, being a good choice for parameter fitting. Therefore, these approximation-based methods may perform better on high-dimensional and large-scale optimization problems.

However, these methods come with significant computational demands and the risk of converging to local minima, especially in complex parameter spaces. The accuracy of these methods is significantly dependent on the quality of initial parameter estimates. Thus, poor initialization potentially results in suboptimal solutions.

From the literature, it can be found that NLS methods are effective for T1ρ mapping but are hampered by slow computational speeds. As a result, NLS is typically applied only to specific regions of interest (ROIs), such as cartilage voxels in the knee joint. NLS can also be unstable when dealing with multi-component relaxation. To address these issues, researchers have developed variants like non-negative least squares (NNLS) [16,17,18] and regularized NLS (RNLS) [18,19].

NNLS adds a non-negativity constraint to stabilize estimations, ensuring real-valued, monotonically decreasing relaxations [16,17,18]. RNLS extends NLS by incorporating regularization penalties to improve stability and accommodate independent (in-voxel) or multi-voxel (spatial) regularization functions [18,19]. Thus, RNLS is more suitable for recovering all voxels in imaging applications. Improved stability makes RNLS somewhat faster than NLS, but it still requires many iterations.

### 1.1. Research Motivation

**Figure 1:**
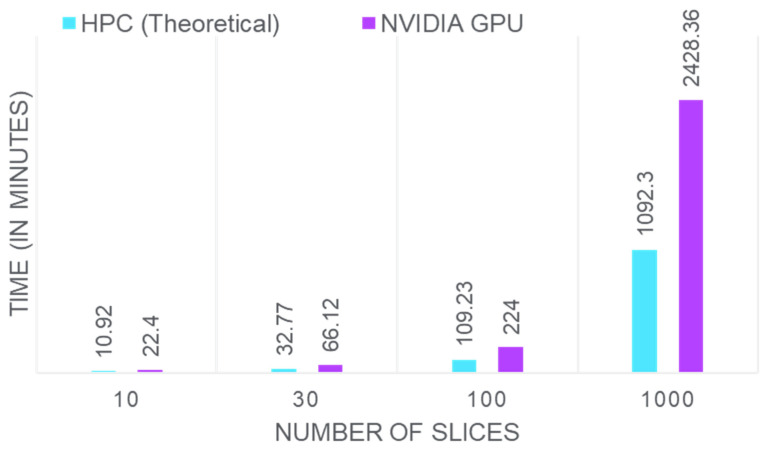
Computational time for NLS fitting across different MRI slice counts on HPC (theoretical) and NVIDIA GPU systems.

Considering an MR image slice of size 256 × 256 = 65,536 voxels, NLS fitting requires approximately 1 millisecond per voxel on high-performance computing (HPC) systems. Thus, fitting all 65,536 voxels would take about 65.54 s for a single slice. Extending this to 4D-MRI data or higher-dimensional scans, such as 512 × 512 or 1024 × 1024, results in exponential increases in computational time. Figure 1 illustrates the time required by our NLS fitting implementation for varying numbers of MRI slices on HPC systems and NVIDIA GPUs. For a single slice, the theoretical time on HPC systems is 1.09 min, while the NVIDIA GPU takes 2.24 min. Assuming a linear increase in time with the number of slices in a dataset, one can easily reach the HPC requirement of 1092.3 min and the NVIDIA GPU requirement of 2240 min for 1000 images. These data highlight the substantial computational demands of NLS fitting.

Due to the time-consuming nature of NLS methods, they are often limited to knee cartilage ROIs. However, recent research highlights the importance of the relaxation map of the full knee joint MRI for assessing osteoarthritis (OA) progression and detecting subtle changes in tissue properties beyond just the cartilage [20]. Therefore, researchers have started exploring artificial intelligence (AI) models as faster alternatives to NLS.

### 1.2. AI-Based Exponential Fitting

Recent advancements in AI offer suitable alternatives to traditional NLS methods. AI models, such as random forest regression, neural networks (NNs), and deep NNs have been extensively utilized to enhance exponential model fitting [21,22,23,24,25]. These models benefit from rapid training and deployment, thus providing stable and accurate curve fitting that is usually faster than existing NLS methods [26,27,28]. By utilizing advanced function approximation abilities, AI models can efficiently address the issues of slow convergence and suboptimal solutions associated with traditional NLS methods [23,24,25,26].

By incorporating AI into the fitting process, researchers have achieved significant improvements in parameter mapping [29,30,31,32]. Additionally, AI models can be trained in an end-to-end manner, i.e., learning optimal mappings from raw k-space or undersampled multi-contrast MRI data to T1ρ maps without the need for explicit MRI reconstruction and NLS fitting [33].

Therefore, researchers are actively exploring faster methods for obtaining quantitative MRI values of the knee joint, including deep learning (DL) approaches [34,35,36,37,38,39,40,41]. However, most supervised DL methods [34,35,36,37] rely on reference values produced by NLS methods. This requires applying NLS to thousands of voxels in each 3D image, and this process must be repeated for every image in a large dataset.

Moreover, the integration of AI-based models with traditional NLS methods has led to the development of hybrid approaches that utilize the strengths of both methods. For example, DL models can provide initial parameter estimates or refine results from NLS methods, thus enhancing accuracy and robustness in T1ρ estimation.

Table 1 presents a description of existing AI-based exponential fitting methods. The table highlights that the majority of AI-based solutions predominantly focus on ME fitting, leaving SE and BE relaxation models relatively underexplored. This limitation creates a significant gap in addressing the diverse relaxation dynamics present in different tissues. Additionally, most existing approaches are often sensitive to noise and prone to inaccuracies in parameter estimation, not providing similar goodness-of-fit properties to NLS-based techniques.

This gap underscores the importance of hybrid approaches, which integrate the strengths of DL and NLS. Such integration ensures improved parameter estimation, enhanced robustness to noise, and better handling of complex multi-component T1ρ mapping tasks. Thus, Table 1 further emphasizes the need for methods that go beyond ME fitting and utilize hybrid strategies to overcome the limitations of AI-based or traditional approaches.

### 1.3. Proposed Solution

Although AI-based exponential fitting models have demonstrated remarkable performance, most researchers have primarily focused on mono-exponential (ME) fitting, with limited exploration of bi-exponential (BE) and stretched-exponential (SE) fitting. Additionally, many AI-based exponential fitting methods require actual MRI data as a reference for training. To address these gaps, this paper proposes the HDNLS, a hybrid model for fast multi-component fitting, dedicated in this work to T1ρ mapping of the knee joint. In HDNLS, we initially designed a DL model that uses synthetic data for training, thus eliminating the need for reference MRI data. However, the estimated map may be sensitive to noise, particularly in the short component of the BE model. To address this, the results from the proposed DL model are further refined using NLS with an optimized number of iterations. To optimize the number of iterations, this paper presents an ablation study in which we analyze the trade-off between the number of NLS iterations and the mean normalized absolute difference (MNAD), as well as between the NLS iterations and normalized root-mean-squared residuals (NRMSRs).

### 1.4. Contributions

The key contributions of the paper are as follows:

(a) Initially, a DL-based multi-component T1ρ mapping method is proposed. This approach utilizes synthetic data for training, thereby eliminating the need for reference MRI data.
(b) The HDNLS method is proposed for fast multi-component T1ρ mapping in the knee joint. This method integrates DL and NLS. It effectively addresses key limitations of NLS, including sensitivity to initial guesses, poor convergence speed, and high computational cost.
(c) Since HDNLS’s performance depends on the number of NLS iterations, it becomes a Pareto optimization problem. To address this, a comprehensive analysis of the impact of NLS iterations on HDNLS performance is conducted.
(d) Four variants of HDNLS are suggested that balance accuracy and computational speed. These variants are Ultrafast-NLS, Superfast-HDNLS, HDNLS, and Relaxed-HDNLS. These methods enable users to select a suitable configuration based on their specific speed and performance requirements.

## 2. Proposed Methodology

The measured signal s of the relaxation of a voxel in position x at a TSL specified by t can be expressed as follows:

(1) $$s\left(x,t\right)=f\left(x,t,\theta \right)+v\left(x,t\right),$$

where v is the noise and $f\left(x,t,\theta \right)$ is the relaxation model with parameters θ. For simplicity, we will omit the spatial position x, assuming that it corresponds to the relaxation process in one voxel. Note that the parameters θ are also related to that specific voxel.

### 2.1. Multi-Component Relaxation Models

Generally, ME, BE, and SE models are commonly used for fitting a T1ρ map. Table 2 presents the mathematical formulations, associated parameters, descriptions, and applicable ranges for these three models. The ME model assumes a homogeneous tissue environment, while the BE model introduces two compartments with distinct relaxation times, and the SE model accounts for heterogeneous environments with distributed relaxation rates.

Table 3 highlights the key features, advantages, and disadvantages of each multi-component relaxation model. The choice of the specific model depends on the tissue characteristics and the requirements of the analysis. While the ME model is simple and suitable for homogeneous tissues, the BE model provides a more detailed representation of tissues with two relaxation components but is computationally more demanding. The SE model offers a balance by modeling complex tissue environments with fewer parameters and greater stability, though its physical interpretation can be less intuitive.

**Figure 2:**
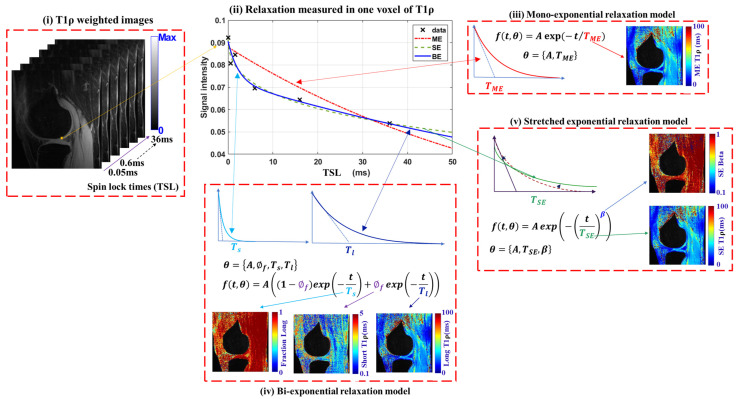
Representative visualization of multi-component T1ρ relaxation models in the knee joint.

Figure 2 illustrates the working of multi-component relaxation models and their respective outputs. It includes T1ρ-weighted images with six TSLs and voxel-wise relaxation measurements. It presents the expected T1ρ maps obtained from ME, BE (the long component, the fraction of the long component, and the short component), and SE models (SE-T1ρ map and beta (β) parameter).

The NLS approach fits the parameters voxel-wise, minimizing the squared Euclidean norm of the residual, using the following:

(2) $$\hat{\theta }=\underset{\theta }{\mathrm{arg min}}\sum_{t=1}^{T}{\left|s\left(t\right)-f\left(t,\theta \right)\right|}^{2},$$

where T is the number of TSLs. This is solved using iterative algorithms such as the one described in [9].

### 2.2. Deep Learning-Based Multi-Component Relaxation Models

**Figure 3:**
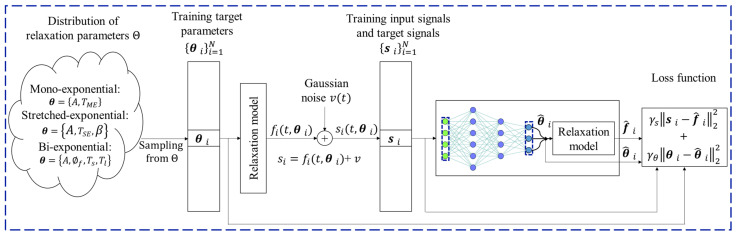
Voxel-by-voxel framework of the DL model for fitting multi-component T1ρ relaxation models with a customized loss function.

Figure 3 shows a diagram of the voxel-by-voxel working of the DL model for fitting multi-component T1ρ relaxation models. Typically, NNs are trained by minimizing the squared error of the target parameters. However, to achieve data consistency, as in NLS estimations, we used a customized loss function, designed as follows:

(3) $$Loss=\sum_{i}\gamma_{s}\;{\left\|s_{i}-{\hat{f}}_{i}\right\|}^{2}+\gamma_{\theta }{\left\|\theta_{i}-{\hat{\theta }}_{i}\right\|}^{2},$$

where i is the index of the data elements in the training dataset, ${\hat{\theta }}_{i}=F_{w}\left(s_{i}\right)$, $s={\left[s\left(1\right)\ldots s\left(T\right)\right]}^{T}$ being a column vector with the measured relaxation signal and $F_{w}$ the fitting network with learning parameters w, and ${\hat{f}}_{i}$ is the relaxation signal produced with the estimated parameters ${\hat{\theta }}_{i}$, using any of the equations presented in Table 2. Target parameters $\theta_{i}$ are sampled from a known distribution, Θ, and $s_{i}$ are synthetically generated using Equation (1) and equations presented in Table 2 and the sampled parameters.

The first term of the loss function tries to reduce the residue, as in NLS approaches, producing a relaxation curve consistent with the synthetically generated measured values at the voxel. The second term tries to reduce the error against the ground truth values. This term also acts as a regularization in the NLS sense. We used $\gamma_{s}=10$ and $\gamma_{\theta }=1$.

The NN architecture comprises 7 repeated blocks of fully connected layers, each containing 512 intermediate elements followed by a non-linear activation function and dropouts. Thereafter, we utilize customized layers to evaluate the relaxation values for the customized loss, as described in Equation (3). Finally, the regression layer is used to predict the T1ρ maps. This network estimates the relaxation parameters voxel-wise, like any of the NLS-based approaches.

### 2.3. Sensitivity Analysis of DL Model

We performed a sensitivity analysis of the proposed DL model to determine the optimal configuration of hyperparameters. Initially, multiple trial-and-error experiments were conducted using various hyperparameter values commonly reported in the literature. The final configuration comprised 7 fully connected layers, each containing 512 intermediate elements. Five different activation functions were also evaluated, such as the Gaussian Error Linear Unit (GELU), Leaky ReLU, the hyperbolic tangent (Tanh), Swish, and ReLU. The model was trained using the Adam optimizer, with a learning rate initialized at 1.0 × 10^−3^, over a maximum of 100 epochs. Each epoch processed mini-batches of 200 samples. We selected $\gamma_{s}$ = 10 and $\gamma_{\theta }$ = 1 as the optimal weights for the proposed customized loss function. During the sensitivity analysis, we varied only the specific parameter under evaluation while keeping all other parameters fixed.

**Figure 4:**
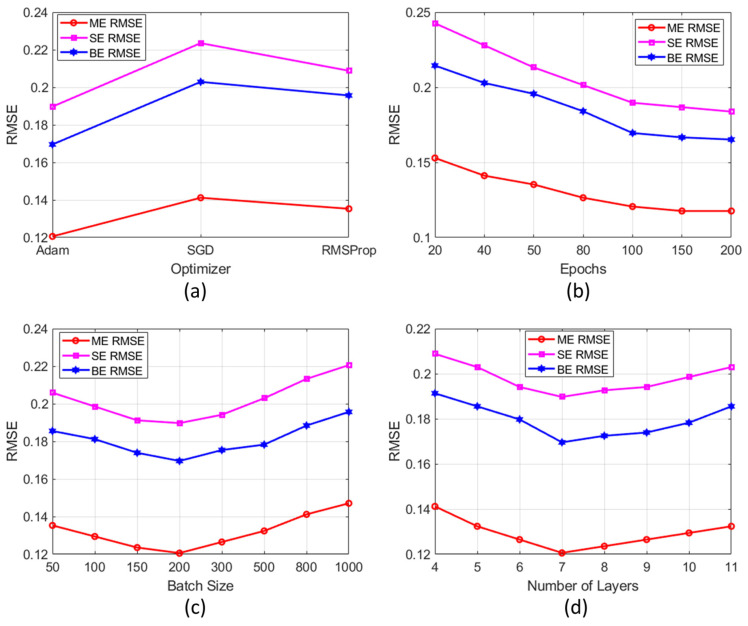
Sensitivity analysis of the DL model: (**a**) optimizer selection, (**b**) epochs, (**c**) batch size, and (**d**) number of layers, on RMSE.

Figure 4 illustrates the impact of (a) optimizer selection (Adam, SGD, and RMSProp), (b) number of epochs (20 to 200), (c) batch size (50 to 1000), and (d) number of layers (4 to 11). From Figure 4a, it is evident that the RMSE shows a decreasing trend as the number of epochs increases. It eventually stabilized around 100 epochs. Adam outperformed SGD and RMSProp by achieving significantly lower RMSE values (see Figure 4b). Smaller batch sizes achieved lower RMSE values, with performance stabilizing beyond a batch size of 200 (see Figure 4c). Finally, seven fully connected layers achieved noticeably lower RMSE values compared to other configurations (see Figure 4d).

**Figure 5:**
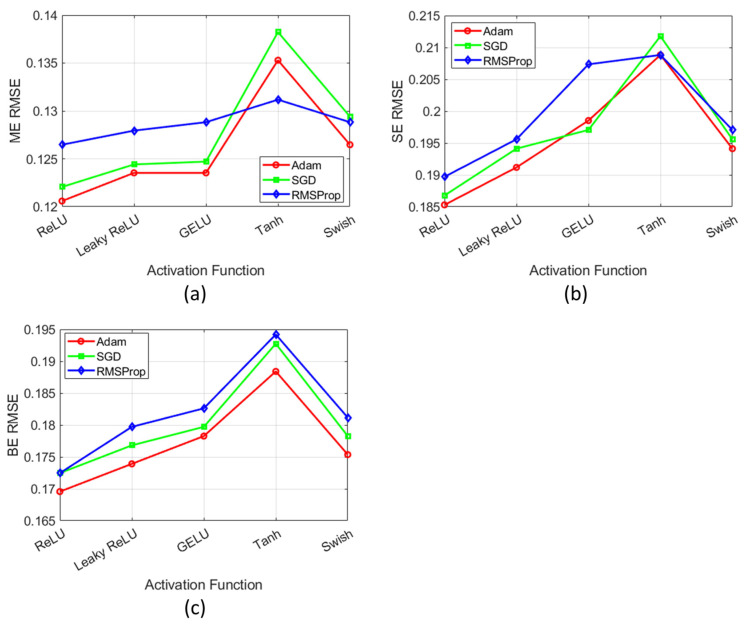
Sensitivity analysis of the combination of activation functions (ReLU, Leaky ReLU, GELU, Tanh, and Swish) with optimizers (Adam, SGD, and RMSProp) for the DL model: (**a**) ME component, (**b**) SE component, and (**c**) BE component.

Figure 5 presents the sensitivity analysis of activation functions (ReLU, Leaky ReLU, GELU, Tanh, and Swish) for the DL model. The analysis evaluated these functions across three optimizers, namely, Adam, SGD, and RMSProp. It showed RMSE variations for ME, SE, and BE components under different activation function–optimizer combinations. Among these, the Adam optimizer combined with the ReLU activation function achieved significantly lower RMSE values across all ME, SE, and BE components.

**Figure 6:**
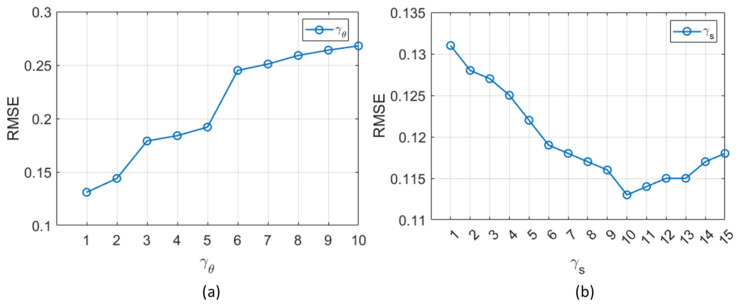
Sensitivity analysis of the loss function with varying $\gamma_{s}$ and $\gamma_{\theta }$ on validation data. (**a**) Impact of $\gamma_{\theta }$ on RMSE with $\gamma_{s}$ fixed at 1, demonstrating an increase in RMSE as $\gamma_{\theta }$ rises from 1 to 10. (**b**) Influence of $\gamma_{s}$ on RMSE with $\gamma_{\theta }$ fixed at 1, showing decreasing RMSE values as $\gamma_{s}$ increases from 1 to 15. However, RMSE starts to increase beyond $\gamma_{s}$ = 10.

Finally, the sensitivity analysis was conducted to evaluate the impact of varying $\gamma_{s}$ and $\gamma_{\theta }$ in the customized loss function on validation data. For better analysis, we evaluated the average RMSE values across all ME, SE, and BE components. Figure 6a shows that increasing $\gamma_{\theta }$ from 1 to 10, with $\gamma_{s}\;$= 1, results in a rise in RMSE. This emphasizes the importance of keeping $\gamma_{\theta }$ relatively low. In contrast, Figure 6b demonstrates a decreasing trend in RMSE as $\gamma_{s}$ increases from 1 to 15, with $\gamma_{\theta }$ = 1. This indicates the benefits of assigning a higher weight to $\gamma_{s}$. However, continually increasing $\gamma_{s}$ in the loss function places an increasingly higher emphasis on minimizing the discrepancy between the measured relaxation signal $s_{i}$ and the model-predicted signal ${\hat{f}}_{i}$. While this might improve the consistency of the relaxation curve with the synthetic measurements, it can increase the error in the estimated parameters. The model prioritizes signal fitting at the expense of accurately estimating the parameters $\theta_{i}$, as the second term in the loss function becomes relatively low, reducing the impact of the regularization term in stabilizing the parameter estimation. As shown in Figure 6b, RMSE increases when $\gamma_{s}$ exceeds 10, indicating a poor balance between signal consistency and parameter accuracy. Based on these observations, we selected $\gamma_{s}$ = 10 and $\gamma_{\theta }$ = 1 as the optimal weights for our loss function.

### 2.4. Training and Validation Analysis

To ensure a robust evaluation of the proposed DL model, the synthetic dataset was divided into three subsets: training, validation, and test data, in an 80:10:10 ratio. The training subset (80% of the data) was used to train the DL model and optimize its parameters. To prevent overfitting, the validation subset (10% of the data) was employed to monitor the model’s performance during training and fine-tune hyperparameters.

**Figure 7:**
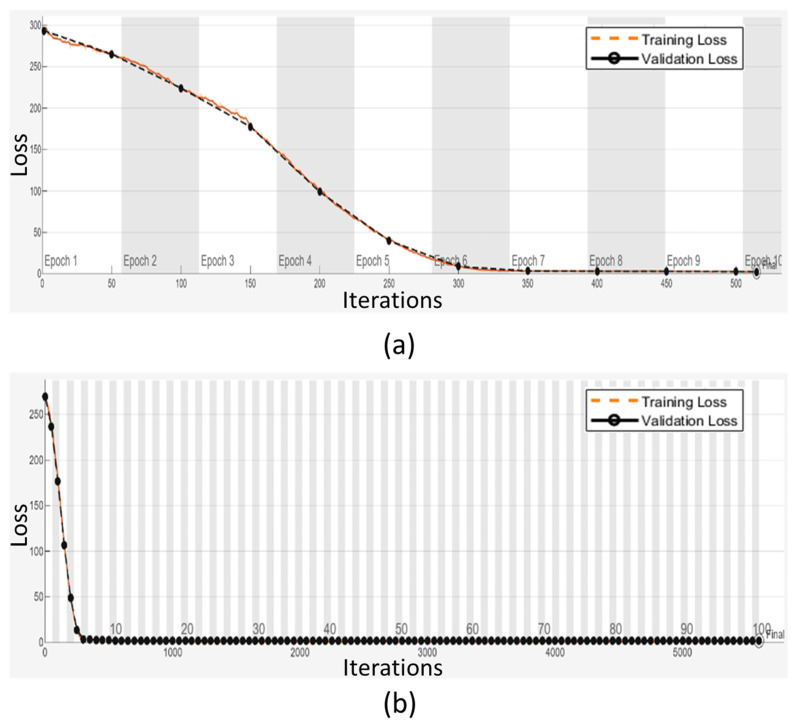
Training and validation loss analysis: (**a**) for 10 epochs and (**b**) for 100 epochs.

Figure 7 illustrates the training and validation loss curves for 10 and 100 epochs, respectively. In Figure 7a, the difference between training and validation loss values is more apparent, reflecting the initial stages of model training. In contrast, Figure 7b shows that, with 100 epochs, it is difficult to distinguish between the loss values for both training and validation converge. This convergence indicates that the DL model does not suffer from overfitting.

This partitioning strategy also ensures efficient training of the DL model while providing a fair and unbiased assessment of its generalization capability. Data for each subset were randomly selected in a stratified manner. Therefore, the used data also preserved the distribution of key features across all subsets. This approach guarantees consistency and reliability in the evaluation process.

Finally, the test subset, comprising 10% of the data, was reserved for the final evaluation of the model’s performance on unseen data.

### 2.5. HDNLS

**Figure 8:**
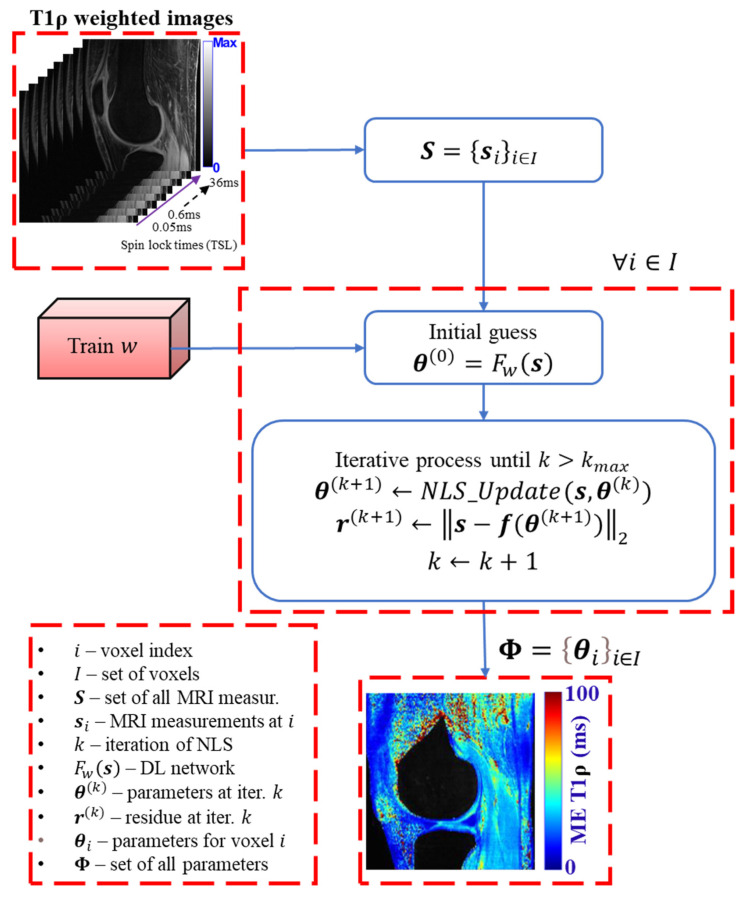
Proposed HDNLS-based fitting of multi-component T1ρ relaxation models in the knee joint.

Figure 8 illustrates the proposed HDNLS-based fitting of multi-component T1ρ relaxation models. The estimated map obtained from the DL model may be sensitive to noise, particularly in the short component of BE. To mitigate this issue, the results from the DL model are further refined using NLS iterations. The DL results serve as an initial guess for NLS. This helps to address both the initial guess dependency and poor convergence speed associated with NLS.

In this paper, TRCG [9,51] is used to solve the NLS problem. At each iteration k, the parameters are estimated using the following:

(4) $$\theta^{\left(k+1\right)}\leftarrow NLS\_Update\left(s,\theta^{\left(k\right)}\right)$$

This update aims to minimize the residuals as follows:

(5) $$r^{\left(k+1\right)}\leftarrow {\left\|s-f\left(\theta^{\left(k+1\right)}\right)\right\|}_{2}$$

where s represents the observed data and $f(\theta^{\left(k+1\right)})$ denotes the model predictions based on the updated parameters. TRCG ensures that each step effectively refines the parameter estimates. Thus, it enhances the convergence of the optimization process to achieve an optimal fit to the data.

### 2.6. T1ρ-MRI Data Acquisition and Fitting Methods

All the experiments were performed on a 3T MRI scanner with magnetization-prepared angle-modulated partitioned k-space spoiled gradient echo sequence snapshots (MAPSS) [52], using six spin-lock time (TSL) values: 0.05, 0.6, 1.6, 6, 16, and 36 ms. We scanned a total of 12 healthy volunteers, with a mean age of 38 and SD = 12.4 years old (n = 7 males and n = 5 females). The MRI scans had a voxel size of 0.7 mm × 0.7 mm × 4 mm, with an FOV of 180 mm × 180 mm × 96 mm. This study was approved by the Institutional Review Board (IRB) of New York University Langone Health and was compliant with the Health Insurance Portability and Accountability Act (HIPAA). All volunteers provided their consent before MRI scanning.

For NLS-based fitting, we used 2000 iterations of the TRCG method [51]. The ME model assumes that the T1ρ values can be in the range of 1–300 ms. The SE model assumes that the T1ρ values can be in the range of 1–300 ms and that β can be in the range of 0.1–1. Because we observed no ME relaxation below 5 ms, we assume that the long component of the BE model can be in the range of 5–300 ms, the short component in the range of 0.5–4 ms, and the BE fraction in the range of 0.01–0.99.

The DL model is trained using the Adam optimizer with a maximum of 100 epochs, and each epoch processes mini-batches of 200 samples. The initial learning rate is set to 1.0 × 10^−3^. The dropout value is set to be 0.1. The proposed model was tested on 3D-T1ρ MRI data from five different healthy subjects. The DL model was trained on 5 healthy datasets.

### 2.7. Performance Metrics

For comparative analysis, four performance metrics were employed: the median of normalized absolute difference (MNAD), normalized root-mean-squared residuals (NRMSRs), fitting time, and normalized root-mean-squared error (NRMSE). Table 4 provides detailed descriptions and mathematical formulations of these metrics.

## 3. Optimal Selection of NLS Iterations for HDNLS

The main objective of this section is to evaluate and compare the performance metrics of NLS and HDNLS on synthetic data by varying the number of NLS iterations. Specifically, the focus is on assessing the convergence speed of both methods and determining whether HDNLS can achieve similar performance to NLS with improved speed.

**Figure 9:**
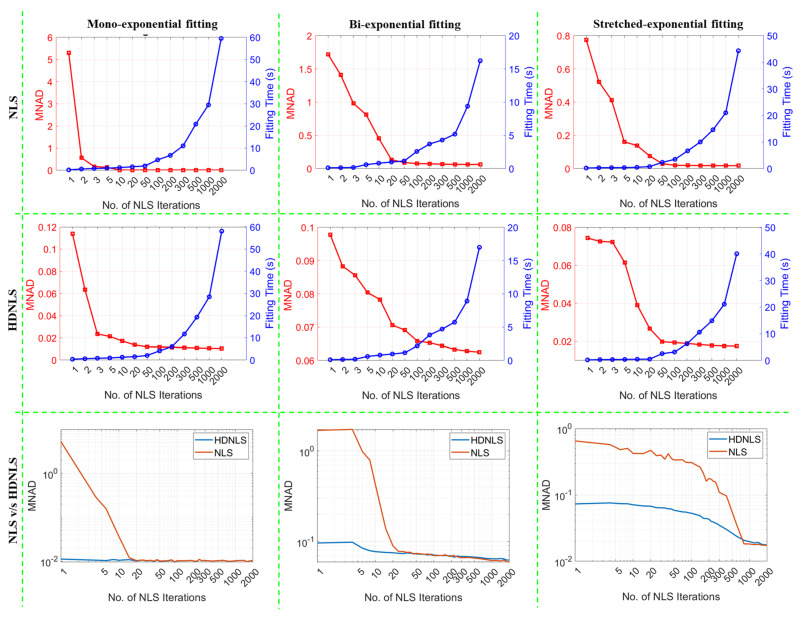
Comparison of NLS and HDNLS across mono-exponential, bi-exponential, and stretched-exponential fitting models on synthetic data. Row 1 illustrates the trade-off between fitting time and MNAD for NLS. Row 2 shows the trade-off between fitting time and MNAD for HDNLS. Row 3 shows the convergence of NLS and HDNLS in terms of MNAD.

Figure 9 shows the comparison between NLS and HDNLS on synthetic data in terms of MNAD across three different fitting models, namely, ME, SE, and BE. Row 1 shows the trade-off between fitting time (blue) and MNAD (red) for NLS as the number of NLS iterations increases. For each fitting model, MNAD decreases initially with more iterations but eventually shows a minor reduction, while the fitting time increases at a rapid rate. Row 2 shows the performance trade-off between fitting time and MNAD for the HDNLS approach. It also shows that as the number of NLS iterations increases, the HDNLS achieves significantly lesser MNAD values. However, after 500 iterations, the improvement in MNAD becomes marginal compared to the increase in fitting time. Row 3 provides direct comparisons between NLS and HDNLS. It shows that HDNLS takes significantly less time compared to NLS when the number of NLS iterations is less than 300. Thereafter, both NLS and HDNLS show almost similar MNAD values with no significant difference.

**Figure 10:**
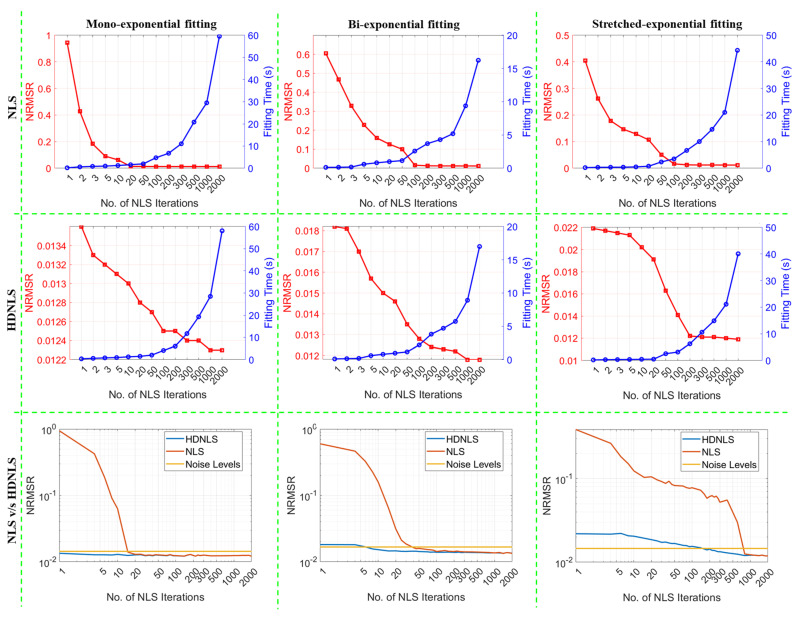
Performance metrics comparison of NLS and HDNLS in terms of fitting time and normalized root-mean-squared residuals (NRMSRs) across ME, SE, and BE fitting models. Row 1 shows the trade-off between fitting time and NRMSR for NLS. Row 2 shows the trade-off between fitting time and NRMSR for HDNLS. Row 3 presents the convergence of both NLS and HDNLS with respect to log (NRMSR).

Similarly, Figure 10 presents the performance of NLS and HDNLS across ME, SE, and BE fitting models on synthetic data in terms of NRMSRs. The yellow line represents the NRMSR of noise only. The primary goal of NLS and HDNLS is to reduce the NRMSR to levels just below the noise level. It was found that in the case of HDNLS, there is little or no benefit in exceeding 500 iterations, except for the SE component. Please note that due to the significant variations in the scale of values for both NLS and HDNLS, it becomes difficult to observe changes in NLS when the number of iterations exceeds 100, 500, and 1000, respectively, for ME, BE, and SE components. To better illustrate this, we present the comparison of NRMSRs between NLS and HDNLS in row 3, clearly demonstrating the improved convergence speed of HDNLS over NLS. Additionally, the difference in NRMSRs for HDNLS after 500 iterations is minimal and can be disregarded.

From row 3 of both Figure 9 and Figure 10, it is evident that HDNLS converges significantly faster than standard NLS in terms of MNAD and NRMSRs, respectively. This makes HDNLS an efficient choice for determining the number of NLS iterations based on specific requirements. For scenarios where speed is the highest priority and small performance compromises are acceptable, 10 iterations, referred to as Ultrafast-NLS, provide quick results with minimal computational cost. Superfast-HDNLS, with 50 NLS iterations, strikes a balance between speed and performance, offering a faster alternative with acceptable accuracy. HDNLS with 200 iterations achieves an optimal middle ground, delivering both rapid convergence and higher performance. However, for SE, we observed that the results at 200 iterations are not as desirable as those for ME and BE; therefore, we selected HDNLS with 300 iterations for SE map fitting only.

For those who prioritize performance over speed, 500 iterations, termed Relaxed-HDNLS, provide the best results while tolerating slower computation times. Thus, HDNLS allows users to select a suitable configuration based on their specific speed and performance requirements.

**Figure 11:**
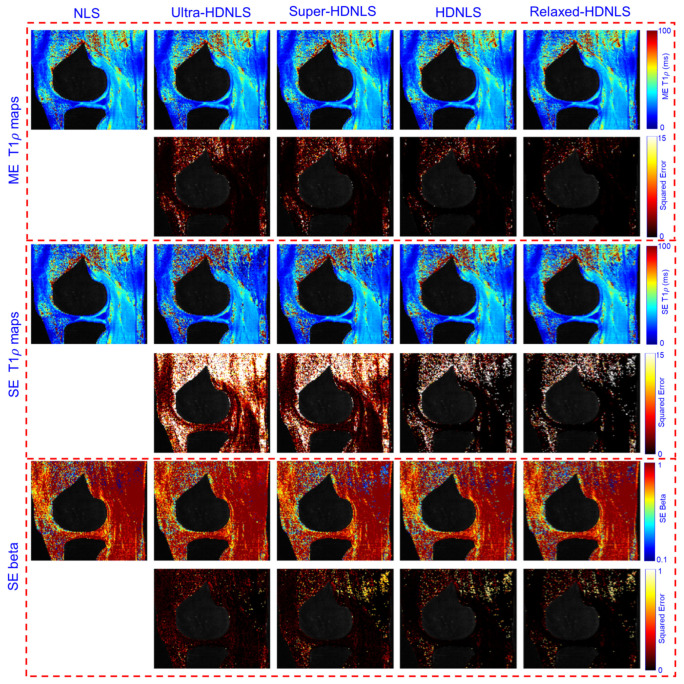
Estimated HDNLS variant-based ME and SE-T1ρ maps for the full knee joint.

**Figure 12:**
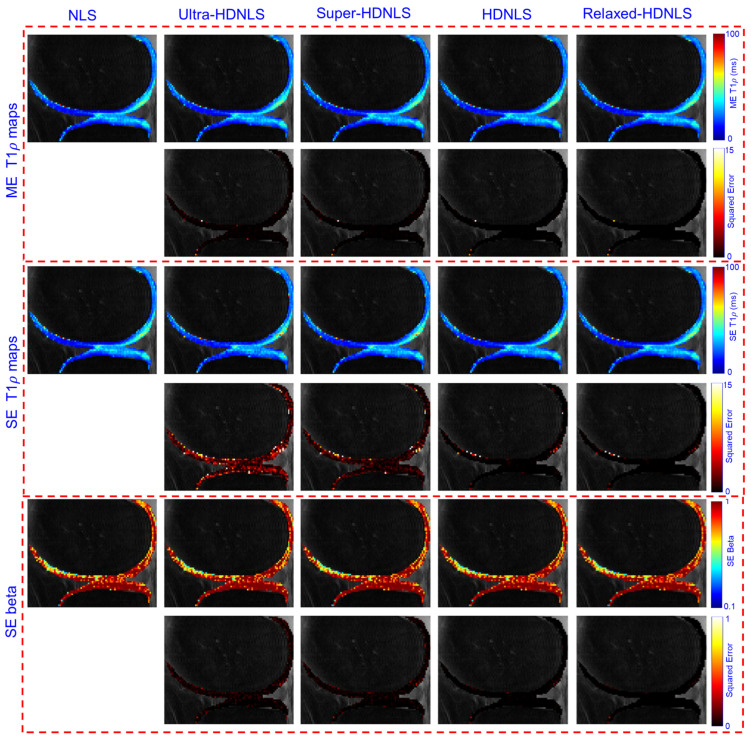
Estimated HDNLS variant-based ME and SE T1ρ maps for knee cartilage ROIs only.

To check the performance of these HDNLS variants, we further tested them on real MRI data (see Section 2.4 for dataset details). Due to the unavailability of ground truth T1ρ maps, we used the NLS-based estimated T1ρ map as the ground truth. The estimated ME and SE T1ρ maps of the whole knee from NLS and HDNLS variants are presented in Figure 11, along with their respective squared errors compared to the NLS-based ME T1ρ map. Similarly, Figure 12 shows the ME and SE T1ρ maps for the knee cartilage only. Both figures demonstrate that HDNLS and Relaxed-HDNLS achieve significantly better results, with notably lower errors, compared to Ultra-HDNLS and Super-HDNLS.

Although it is challenging to visually distinguish differences between the obtained T1ρ maps, the evaluated squared error differences provide a clear distinction. Higher squared error values indicate poorer performance. From Figure 11 and Figure 12, the squared error maps for HDNLS and Relaxed-HDNLS show significantly lower errors. This highlights their superior performance and closer alignment with the NLS-based T1ρ maps.

**Figure 13:**
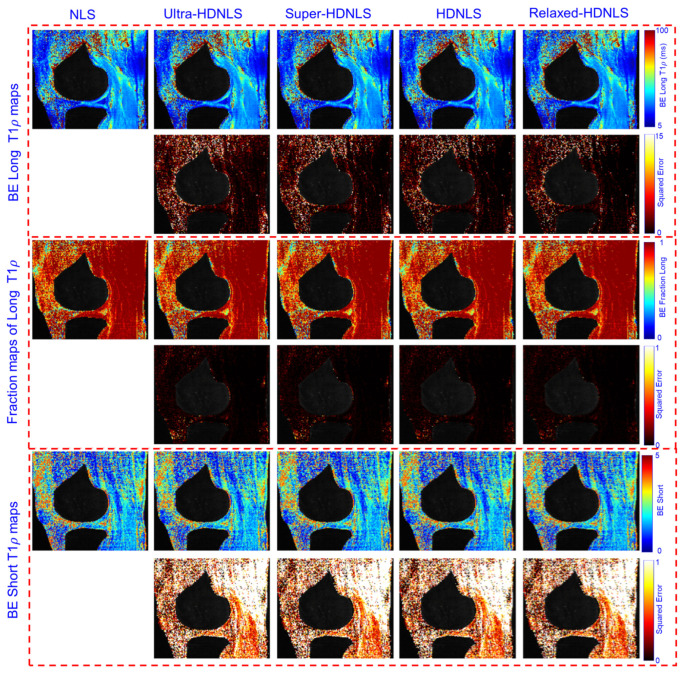
Estimated HDNLS variant-based BE long T1ρ, fraction of the long T1ρ, and short T1ρ maps for the full knee joint.

**Figure 14:**
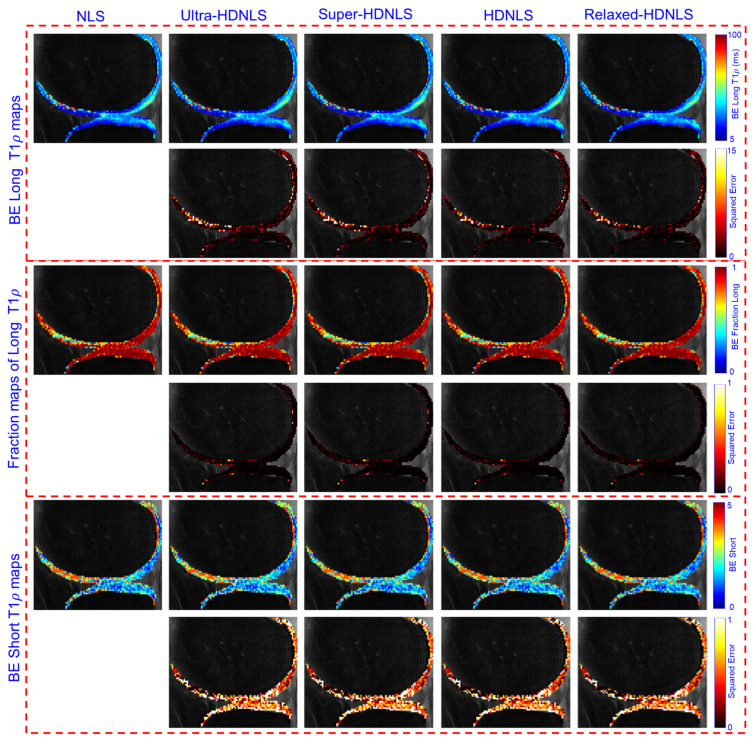
Estimated HDNLS variant-based BE long T1ρ, fraction of the long T1ρ, and short T1ρ maps for knee cartilage ROIs only.

Figure 13 presents the estimated BE long T1ρ component, the fraction of the long T1ρ component, and short T1ρ maps of the whole knee from NLS and HDNLS variants, along with their respective squared errors compared to the ground truth, i.e., the NLS-based ME T1ρ map. Similarly, Figure 14 shows the estimated BE long T1ρ component, the fraction of the long T1ρ component, and short T1ρ maps for knee cartilage only. Both figures demonstrate that HDNLS and Relaxed-HDNLS achieve significantly better results with much lower errors compared to Ultra-HDNLS and Super-HDNLS.

Table 5 and Table 6 provide a quantitative analysis of HDNLS variants, i.e., Ultra-HDNLS, Super-HDNLS, HDNLS, and Relaxed-HDNLS, for estimating ME, SE, and BE T1ρ maps using real data for the full knee and knee cartilage, respectively. The Relaxed-HDNLS variant outperformed the others by achieving the minimum MNAD and NRMSR values. In contrast, Ultra-HDNLS provided the fastest fitting time for the estimation of all ME, SE, and BE T1ρ maps, but had higher MNAD and NRMSR values. Therefore, Table 5 and Table 6 highlight a trade-off in selecting the best approach. Faster fitting times come at the cost of performance. This necessitates careful consideration of whether to prioritize speed or accuracy when estimating T1ρ maps.

## 4. Performance Analysis

Based on the visual and quantitative analysis presented in Section 3, we identified a trade-off in selecting the best approach. Faster fitting times come at the cost of performance, necessitating careful consideration of whether to prioritize speed or accuracy when estimating T1ρ maps. Therefore, we selected an optimized HDNLS variant named HDNLS. For ME and BE, it will use 200 NLS iterations, while for SE, we chose 300 iterations. The idea was to select a solution that is around 30 times faster than NLS with significantly fewer errors on synthetic data. For ME and BE, when NLS iterations were 200, we achieved around a 34-times better fitting speed with comparable performance to NLS. However, with 200 iterations for SE fitting, we observed slightly more errors compared to when NLS iterations were set to 300. Due to this, for SE fitting, HDNLS (NLS iterations = 300) shows a speed improvement approximately 27 times better than NLS, with similar results compared to NLS.

The main objective of this section is to compare the performance of HDNLS against NLS, RNLS, and DL.

**Figure 15:**
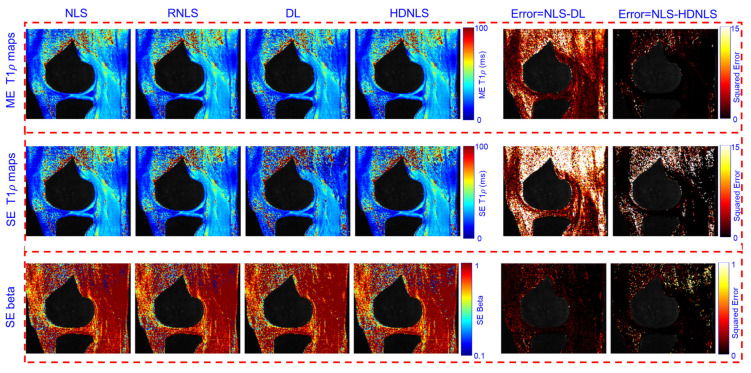
Estimated NLS-, RNLS-, DL-, and HDNLS-based ME and SE T1ρ maps for the full knee joint.

**Figure 16:**
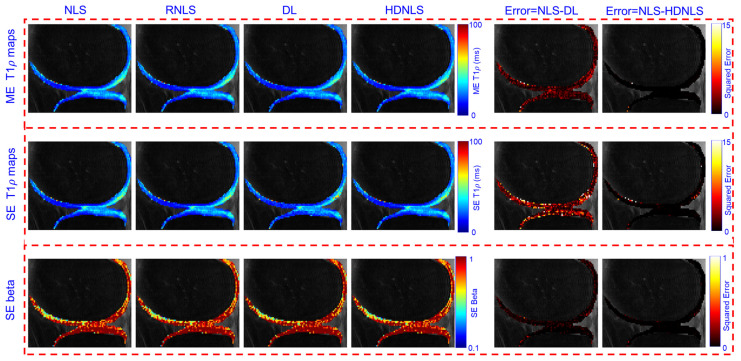
Estimated NLS-, RNLS-, DL-, and HDNLS-based ME and SE T1ρ maps for the knee cartilage ROIs only.

Figure 15 and Figure 16 present the estimated ME and SE T1ρ maps for the full MRI joint and knee cartilage, obtained using NLS, RNLS, DL, and HDNLS, respectively. The error was also computed between NLS and DL and between NLS and HDNLS for the full knee joint and knee cartilage ROIs. The analysis shows that the error between NLS and HDNLS is lower compared to that between NLS and DL. This indicates that, compared to DL, the proposed HDNLS method can more effectively serve as an alternative to NLS and RNLS for estimating ME T1ρ, SE T1ρ, and SE beta maps.

The higher error observed in the BE short component is due to its susceptibility to noise caused by low signal intensity and rapid decay dynamics. This fast decay can lead to insufficient signal sampling, negatively affecting accurate fitting. Furthermore, short components exhibit increased sensitivity to minor variations in model parameters, resulting in escalated errors during the fitting process.

**Figure 17:**
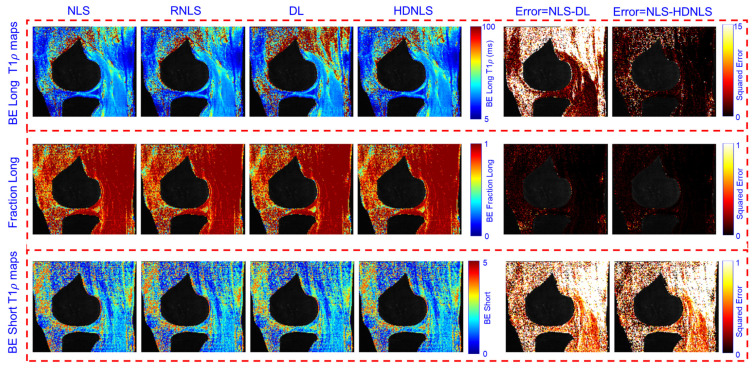
Estimated NLS-, RNLS-, DL-, and HDNLS-based BE long T1ρ component, fraction of the long T1ρ component, and short T1ρ maps for the full knee joint.

**Figure 18:**
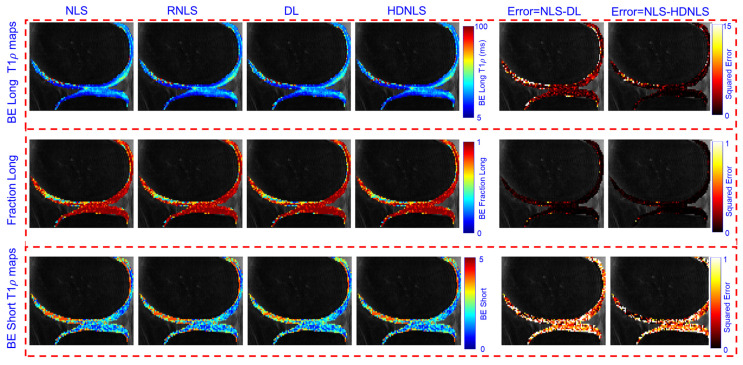
Estimated NLS, RNLS, DL, and HDNLS-based BE long T1ρ component, fraction of the long T1ρ component, and short T1ρ maps for knee cartilage ROIs only.

Figure 17 and Figure 18 show the predicted BE long T1ρ maps, the fraction of the long BE T1ρ component, and the short T1ρ maps for the full knee joint and knee cartilage ROIs, generated using NLS, RNLS, DL, and HDNLS. The error between NLS and DL and between NLS and HDNLS was also calculated for both the full knee joint and knee cartilage ROIs. The analysis demonstrates that the error between NLS and HDNLS is lower than that between NLS and DL. Thus, it was revealed that, compared to DL, the HDNLS method is a more effective alternative to NLS and RNLS for estimating BE components.

Table 7 presents a quantitative analysis of NLS, RNLS, DL, and HDNLS methods for multi-component T1ρ mapping (ME, SE, and BE) on synthetic data in terms of MNAD, fitting time (in seconds), and NRMSR. It indicates that although NLS outperforms RNLS, DL, and HDNLS, the differences with respect to RNSL and HDNLS are not significant. Additionally, DL achieved better speed compared to the other approaches, but it also achieved the poorest performance. Overall, HDNLS achieved comparable performance to NLS and RNLS and significantly better performance than DL.

Table 8 presents a comparison of MNAD and NRMSE values for DL and HDNLS methods for multi-component T1ρ mapping (ME, SE, and BE) on real data for both the full knee joint and knee cartilage ROIs. The results show that HDNLS significantly outperforms DL by achieving lower MNAD and NRMSE values across all ME, SE, and BE categories. This indicates that HDNLS is a better alternative to NLS than DL for T1ρ mapping.

Table 9 presents a computational speed (in seconds) analysis of NLS, RNLS, DL, and HDNLS methods for multi-component T1ρ mapping (ME, SE, and BE) on real data for both the full knee and knee cartilage ROIs. The results indicate that HDNLS significantly outperforms the traditional methods (NLS and RNLS) in terms of computational speed, although it is slower than DL. Though DL demonstrates substantial speed improvements over NLS, RNLS, and HDNLS, it also results in greater error compared to HDNLS (see Table 8). As stated in Section 3, our objective was to find a suitable trade-off between performance and computational speed. Therefore, while HDNLS takes more time than DL, it also shows significant performance improvements over DL. Thus, HDNLS achieves comparable performance to NLS and RNLS. Compared to NLS, it provides approximately 13- to 14-times better speed for each ME, SE, and BE component for the full knee joint. Additionally, for the knee cartilage ROIs, it offers about 11-, 12-, and 11-times better speed than NLS, respectively.

## 5. Discussion, Limitations, and Future Directions

### 5.1. Discussion

The proposed HDNLS method effectively addresses the inherent limitations of traditional NLS [6,7,8,9,10,11,12,13,14,15], NNLS [16,17,18], RNLS [18,19], and AI-based [21,22,23,24,25,26] T1ρ fitting methods by combining their strengths. Since NLS, NNLS, and RNLS approaches are often slow and necessitate careful initialization, HDNLS employs a DL model to provide an initial guess for the NLS fitting process. Additionally, HDNLS eliminates the dependency on reference data, as it was trained on synthetic data. By utilizing DL predictions for NLS initialization, HDNLS achieves superior parameter estimation compared to AI-based models [21,22,23,24,25,26] while avoiding the high computational costs typically associated with conventional methods, such as NLS [6,7,8,9,10,11,12,13,14,15], NNLS [16,17,18], and RNLS [18,19]. Additionally, HDNLS extends its capabilities beyond end-to-end DL models [33,34,35,36,37], which are primarily designed for ME fitting, accommodating various multi-component fitting scenarios such as BE and SE.

As we can see in Table 7, HDNLS significantly outperforms DL by achieving lower MNAD and NRMSE values across all categories for ME, SE, and BE on real data from both the full knee joint and knee cartilage ROIs. Thus, HDNLS is a more robust and superior alternative to DL for accurate T1ρ mapping. However, HDNLS has poorer computational speed than DL due to the use of NLS iterations, as indicated in Table 8. Nonetheless, HDNLS markedly surpasses traditional methods like NLS and RNLS in speed. The rapid speed of DL comes at the cost of accuracy, as reflected in the higher MNAD and NRMSE values compared to HDNLS. Our aim, as outlined in Section 3, was to strike a balance between performance metrics and computational efficiency.

Therefore, we prefer HDNLS over DL, even though it requires more time than DL. HDNLS provides significant performance enhancements, achieving results comparable to those of NLS and RNLS. Specifically, HDNLS achieves approximately 13 to 14 times the speed of NLS across ME, SE, and BE components for full knee joint analysis and offers about 11- to 12-times better speed for knee cartilage ROIs. Therefore, HDNLS stands out as an efficient solution for fast multi-component T1ρ mapping, particularly in knee joint imaging, striking an optimal balance between efficiency and high precision.

### 5.2. Limitations of HDNLS

Although the proposed HDNLS model demonstrates promising performance in T1ρ mapping, it does have certain limitations, which include the following:

(a) The analysis of the BE short component reveals significant challenges due to its susceptibility to noise and rapid decay dynamics. These factors contribute to increased errors in fitting processes, making accurate estimation of BE short relaxation times particularly difficult. The sensitivity of these components to minor parameter variations further complicates the issue.
(b) Despite its computational efficiency compared to traditional NLS and RNLS methods, the iterative nature of HDNLS makes it more computationally demanding than alternative DL approaches.
(c) Although HDNLS demonstrates comparable performance in T1ρ mapping, its applicability to other quantitative MRI parameters, such as T2 mapping or diffusion tensor imaging (DTI), remains unexplored.
(d) The current implementation of HDNLS does not incorporate self-supervised learning techniques. As a result, it may not fully utilize undersampled multi-contrast MRI data.

### 5.3. Future Directions

Although HDNLS has demonstrated remarkable performance, several challenges remain that need to be addressed in the future.

(a) Further studies are essential to evaluate the performance of HDNLS across diverse patient populations and imaging protocols. Testing the model with data from various MRI scanners is crucial. This will help identify potential limitations and improve generalizability in clinical settings.
(b) While HDNLS primarily targets T1ρ mapping, future research could adapt its methodology for other quantitative MRI parameters, such as T2 mapping and diffusion tensor imaging (DTI). This broader application could provide valuable insights into tissue characterization and significantly enhance diagnostic capabilities.
(c) Self-supervised learning techniques could minimize reliance on labeled and synthetic data. This would enhance the training efficiency of HDNLS. Additionally, this approach may improve HDNLS’s ability to learn from raw or undersampled multi-contrast MRI data. Consequently, we can develop an end-to-end HDNLS model for parameter fitting.
(d) Optimizing the DL architecture or incorporating advanced techniques like attention mechanisms or transformer networks could enhance HDNLS’s ability to capture complex data patterns. This improvement would reduce the number of NLS iterations required. As a result, the overall process would become faster. Additionally, using advanced regularization techniques could make HDNLS more robust to noise and outliers. This would ensure more accurate parameter estimation in challenging scenarios.
(e) The BE short component is inherently challenging to estimate due to its rapid decay dynamics and low signal intensity, making it more susceptible to noise. We propose the following steps to address this issue in the near future: (i) Implement regularization techniques, such as spatial smoothness constraints and multi-voxel regularization, to stabilize parameter estimation across neighboring voxels. (ii) Integrate advanced noise-aware DL architectures, incorporating uncertainty quantification, into the DL component of HDNLS. These architectures will explicitly account for input data variability, improving robustness and reliability under noisy conditions. (iii) Explore adaptive weighting schemes in the loss function during training for BE fitting to assign appropriate emphasis to the short component. This approach aims to reduce the sensitivity of the short component to noise while maintaining overall model performance.
(f) HDNLS can be extended using a pre-trained model on similar domains, such as quantitative MRI or exponential model fitting tasks. This may accelerate convergence, improve generalization, and potentially expand the range of applicability for voxel-wise multi-component T1ρ fitting.
(g) As part of future studies, we will evaluate the HDNLS framework in clinical workflows to assess its utility and impact on patient diagnosis and treatment planning.

## 6. Conclusions

This paper introduced HDNLS, a hybrid model that combined DL and NLS for efficient multi-component T1ρ mapping in the knee joint. HDNLS effectively addressed key limitations of NLS, such as sensitivity to initial guesses, poor convergence speed, and high computational costs. By utilizing synthetic data for training of the DL model, we eliminated the need for reference MRI data and enhanced noise robustness through NLS refinements. We conducted a comprehensive analysis of NLS iterations on FastNL and proposed four variants. These variants were Ultrafast-NLS, Superfast-HDNLS, HDNLS, and Relaxed-HDNLS. Among these, HDNLS achieved an optimal balance between accuracy and speed. HDNLS achieved performance comparable to NLS and RNLS with a minimum 13-times increase in fitting speed. Although HDNLS is slower than DL, it significantly outperformed it in terms of MNAD and NRMSE. Therefore, HDNLS serves as a robust and faster alternative to NLS for multi-component T1ρ fitting.

## Figures and Tables

**Table 1:** Comparative analysis of the existing AI-based exponential fitting methods.

Ref.	Year	Method	Features	Open Challenges
[21]	2017	Monte Carlo simulations and a random forest regressor	- Established a robust mapping from diffusion-weighted MR signal features to the underlying microstructure parameters of brain tissue - Estimated water residence time in brain white matter	- Did not estimate multi-component T1ρ map - Did not provide uncertainty estimates for predictions - Used a very basic representation of white matter
[22]	2019	Deep neural network (DNN)	- Employed non-supervised DNNs to fit the Intravoxel Incoherent Motion (IVIM) - Does not rely on priors - Parameter maps were smooth and consistent within homogeneous tissues	- DNN may require retraining for different acquisition protocols - Training and testing on same data - Did not improve Bayesian convergence in low SNR regions
[24]	2020	DNN	- Used a DNN to directly estimate myelin water fraction (MWF) - Can produce whole-brain MWF maps in approximately 33 s - Better voxel-wise agreement was observed between DNN-based MWF estimates and NNLS-based ground truths	- DNN relied on NNLS-generated ground truths, so it inherited the NNLS method’s vulnerability to noisy data - DNNs are sensitive to noise and artifacts in the input data
[33]	2019	MANTIS	- Integrated a model-augmented neural network with incoherent k-space sampling for efficient MR parameter mapping - Combined physics-based modeling with DL for accelerated image reconstruction - Reduced noise and artifacts in reconstructed parameter maps	- Generalization to diverse datasets may need further validation due to dependency on sampling patterns - Performance depends on the quality of its training data, which could lead to overfitting and limited generalizability
[38]	2020	Model-based deep adversarial learning	- Employed a generative adversarial network (GAN) for enhanced reconstruction quality - Achieved rapid parameter mapping with high fidelity and robustness - Effectively removed noise and artifacts	- Adversarial training is computationally demanding and sensitive to hyperparameter tuning - GANs may generate outputs that appear realistic but deviate from the true underlying signal
[39]	2021	MoDL-QSM	- Combined model-based reconstruction with DL for quantitative susceptibility mapping (QSM) - Used iterative model unrolling with DL modules to improve QSM accuracy - Produced high-quality susceptibility maps with reduced streaking artifacts	- Model unrolling introduces computational overhead - Requires careful hyperparameter optimization to ensure convergence
[40]	2021	DOPAMINE	- Integrated model-based and deep learning techniques for fast T1 mapping - Used the variable flip angle (VFA) method for efficient data acquisition - Reduced dependency on large training datasets by incorporating physics-based modeling - Handled noise and artifacts effectively	- Dependent on specific acquisition protocols (VFA), limiting flexibility for other methods - Computationally intensive due to the integration of model-based techniques
[37]	2021	RELAX	- Utilized two physical models, such as the MR imaging model and the quantitative imaging model, in network training to fit the parameters - Eliminated the need for fully sampled reference datasets - Directly reconstructs MR parameter maps from undersampled k-space data	- Tested only on simulated and coil-combined data - Only a residual U-Net was used - RELAX’s performance relies on the precision of its physical models, limiting its robustness to inaccuracies
[35]	2023	SuperMAP	- Achieved mapping with acceleration factors up to R = 32 - Generated relaxation maps from a single scan using optimized data acquisition - Directly converted undersampled parameter-weighted images into quantitative maps - Performed well on both retrospectively undersampled and prospective data, demonstrating adaptability	- Performance relies on the quality and diversity of retrospectively undersampled training data - Heavily reliant on the quality of the undersampling patterns; suboptimal patterns may degrade performance
[41]	2024	RELAX-MORE	- Used self-supervised learning, eliminating the need for large, labeled datasets - Unrolled an iterative model-based qMRI reconstruction into a DL framework - Produced accurate parameter maps that correct image artifacts, remove noise, and recover features even in regions with imperfect imaging conditions	- The unrolling of iterative processes and subject-specific training could still be computationally intensive - Subject-specific training might require more careful convergence monitoring, as the self-supervised framework relies on single-subject data

**Table 2:** Multi-component relaxation models: mono-exponential (ME), bi-exponential (BE), and stretched-exponential (SE) models.

Model	Equation	Parameters θ	Description	Range
ME [20,42,43,44]	$f\left(t,\theta \right)=A\;\exp\left(-\frac{t}{T_{ME}}\right)$	$\left\{A,T_{ME}\right\}$	Assumes tissue is homogeneous with a single relaxation time. A is the complex-valued amplitude and $T_{ME}$ is the real-valued relaxation time.	$T_{ME}>0\;ms$ (for knee cartilage ≥ 5 ms)
BE [45,46,47]	$f\left(t,\theta \right)=A\left((1-\emptyset_{f})\exp\left(-\frac{t}{T_{s}}\right)+\emptyset_{f}\exp\left(-\frac{t}{T_{l}}\right)\right)$	$\left\{A,\emptyset_{f},T_{s},T_{l}\right\}$	Accounts for two compartments with distinct relaxation times, $T_{s}$ (short) and $T_{l}$ (long). $\emptyset_{f}$ is the fraction of the long compartment.	$0{\leq \emptyset }_{f}\leq 1$ $T_{s}<5\;ms$ $T_{l}\geq 5\;ms$
SE [48,49,50]	$f\left(t,\theta \right)=A\exp\left(-{\left(\frac{t}{T_{SE}}\right)}^{\beta }\right)$	$\left\{A,T_{SE},\beta \right\}$	Models heterogeneous environments with distributed relaxation rates. $T_{SE}$ is the relaxation time, and β is the stretching exponent.	0<β≤1 $T_{SE}>0\;ms$

**Table 3:** Features, advantages, and disadvantages of different relaxation models (ME, BE, and SE).

Model	Features	Advantages	Disadvantages
ME	- Fundamental model for single-component relaxation	- Simple and easy to implement - Suitable for homogeneous tissues with a single relaxation component	- Limited in handling tissues with multiple relaxation components
BE	- Extends the ME model by introducing two compartments (short and long) - Represents two-component decay systems - Accounts for two non-exchanging compartments in tissues, each with distinct short and long relaxation times	- Better representation of tissues with two relaxation components, e.g., free water and water bonded to macromolecules	- Requires more parameters and is computationally expensive - May face convergence issues during fitting - The short component is usually sensitive to noise
SE	- Reduces to ME when β = 1 - Can be used as an alternative to BE for describing tissue heterogeneity - Can approximate a broad distribution of relaxation rates with fewer parameters, simplifying complex tissue modeling	- Suitable for heterogeneous tissues - More stable and requires fewer parameters than BE - Can describe multi-component relaxation	- Less straightforward physical interpretation compared to BE

**Table 4:** Mathematical formulations of performance metrics used for comparative analysis.

Metric	Description	Equation	Symbols
MNAD	Measures the median of the normalized absolute difference between estimated and reference T1ρ parameters across voxels.	$MNAD(\Phi )=\underset{i\in I,p\in P}{\mathrm{median}}\left(\frac{\left|{\bar{\theta }}_{i,p}-\theta_{i,p}\right|}{\left|{\bar{\theta }}_{i,p}\right|}\right)$	$\theta_{i,p}$ and ${\bar{\theta }}_{i,p}$ are the estimated and reference parameters *p* of the voxel indexed by *i* that belongs to the region of interest or set of voxels *I*, and $\Phi ={\left\{\theta_{i}\right\}}_{i\in I}$.
NRMSR	Assesses the size of the residual between MR measurements and predicted MR signals.	$NRMSR(F)=\sqrt{\underset{i\in I}{mean}\frac{{\left\|s_{i}-{\hat{f}}_{i}\right\|}_{2}}{{\left\|s_{i}\right\|}_{2}}}$	$s_{i}$ is the measured MR signal at the voxel indexed by *i*, and ${\hat{f}}_{i}$ is the predicted MR signal at the same voxel with $\theta_{i}$$,\;\mathrm{and}\;F={\left\{{\hat{f}}_{i}\right\}}_{i\in I}$.
NRMSE	Assesses the normalized value of the root-mean-squared error (RMSE) across the reference parameters.	$\begin{array}{c} NRMSE\left(\Phi \right)=\\ \sqrt{\frac{\underset{i\in I,p\in P}{mean}{\left|{\bar{\theta }}_{i,p}-\theta_{i,p}\right|}^{2}}{\underset{i\in I,p\in P}{mean}{\left|{\bar{\theta }}_{i,p}\right|}^{2}}}\# \end{array}$	$\theta_{i,p}$ and ${\bar{\theta }}_{i,p}$ are the estimated and reference parameters *p* of the voxel indexed by *i* that belongs to the region of interest or set of voxels *I*, and $\Phi ={\left\{\theta_{i}\right\}}_{i\in I}$.
Fitting Time	Represents the time required by models (e.g., NLS, RNLS, HDNLS, and DL) to compute the T1ρ map for one MRI slice, measured on the same hardware.	Fitting Time=toc−tic	tic: Start time of the fitting process. toc: End time of the fitting process.

**Table 5:** Quantitative analysis of HDNLS variants for ME, SE, and BE T1ρ maps on real data for full knee joints. Bold quantitative values indicate the outperforming parameter for the specific model.

Method	Metric	Ultra-HDNLS	Super-HDNLS	HDNLS	Relaxed-HDNLS
ME	MNAD (%)	4.68	4.22	4.03	**3.82**
	Fitting time (s)	**0.99**	1.76	5.13	10.96
	NRMSR	0.03	0.03	0.02	**0.02**
SE	MNAD (%)	13.49	8.92	7.18	**6.75**
	Fitting time (s)	**1.12**	1.85	3.42	7.12
	NRMSR	1.09	0.70	0.18	**0.15**
BE	MNAD (%)	21.38	20.26	19.87	**19.24**
	Fitting time (s)	**1.22**	2.14	3.96	7.73
	NRMSR	0.03	0.03	0.02	**0.02**

**Table 6:** Quantitative analysis of HDNLS variants for ME, SE, and BE T1ρ maps on real data for knee cartilage ROIs only. Bold quantitative values indicate the outperforming parameter for the specific model.

Method	Metric	Ultra-HDNLS	Super-HDNLS	HDNLS	Relaxed-HDNLS
ME	MNAD (%)	4.36	4.16	3.86	**3.74**
	Fitting time (s)	**0.52**	0.95	2.38	5.73
	NRMSR	0.03	0.02	0.02	**0.02**
SE	MNAD (%)	13.24	8.73	7.12	**6.67**
	Fitting time (s)	**0.51**	0.87	2.28	3.71
	NRMSR	1.09	0.70	0.18	**0.14**
BE	MNAD (%)	20.34	19.77	19.28	**18.94**
	Fitting time (s)	**0.46**	0.87	1.85	3.26
	NRMSR	0.03	0.02	0.02	**0.02**

**Table 7:** Quantitative analysis of NLS, RNLS, DL, and HDNLS for ME, SE, and BE T1ρ maps on synthetic data. Bold quantitative values indicate the outperforming parameter for the specific model.

Method	Metric	NLS	RNLS	DL	HDNLS
ME	MNAD (%)	**3.65**	3.69	4.29	3.70
	Fitting time (s)	37.48	32.83	**0.79**	4.94
	NRMSR	**0.02**	0.02	0.03	0.02
SE	MNAD (%)	**6.18**	6.27	7.61	6.32
	Fitting time (s)	41.9	37.75	**0.61**	4.17
	NRMSR	**0.15**	0.15	0.18	0.16
BE	MNAD (%)	**16.19**	16.36	18.37	16.47
	Fitting time (s)	11.73	11.16	**0.52**	2.48
	NRMSR	**0.02**	0.02	0.02	0.02

**Table 8:** Quantitative analysis of NLS, RNLS, DL, and HDNLS for ME, SE, and BE T1ρ maps on real data for both full knee joint and knee cartilage ROIs only. Bold quantitative values indicate the outperforming parameter for the specific model.

Method	Metric	Full Knee		Knee Cartilage	
		DL	HDNLS	DL	HDNLS
ME	MNAD (%)	22.36	**18.93**	19.32	**17.20**
	NRMSE (%)	24.05	**21.25**	21.20	**19.31**
SE	MNAD (%)	26.38	**23.94**	23.38	**21.64**
	NRMSE (%)	25.81	**22.94**	22.46	**19.85**
BE	MNAD (%)	17.70	**13.02**	14.09	**12.24**
	NRMSE (%)	20.16	**16.36**	18.37	**14.85**

**Table 9:** Computational speed analysis of NLS, RNLS, DL, and HDNLS for ME, SE, and BE T1ρ maps on real data for both full knee joint and knee cartilage only. Bold quantitative values indicate the outperforming parameter for the specific model.

Method	Metric	NLS	RNLS	DL	HDNLS
ME	Full Knee	274.68	234.22	**1.35**	19.32
	Knee Cartilage	76.37	65.82	**0.92**	6.78
SE	Full Knee	293.49	262.92	**1.78**	22.50
	Knee Cartilage	91.12	88.39	**1.67**	7.51
BE	Full Knee	257.38	229.13	**1.21**	18.14
	Knee Cartilage	70.26	67.14	**0.84**	6.13

## Data Availability

The data presented in this study are available upon request from the corresponding author. The data are not publicly available due to privacy and ethical policy.
